# *Leishmania* Promastigotes Lack Phosphatidylserine but Bind Annexin V upon Permeabilization or Miltefosine Treatment

**DOI:** 10.1371/journal.pone.0042070

**Published:** 2012-08-01

**Authors:** Adrien Weingärtner, Gerdi Kemmer, Frederic D. Müller, Ricardo Andrade Zampieri, Marcos Gonzaga dos Santos, Jürgen Schiller, Thomas Günther Pomorski

**Affiliations:** 1 Institute of Biology, Humboldt-Universität zu Berlin, Berlin, Germany; 2 Department of Plant Biology and Biotechnology, University of Copenhagen, Frederiksberg C, Copenhagen, Denmark; 3 Institute of Chemistry, Humboldt-Universität zu Berlin, Berlin, Germany; 4 Instituto de Biociências, Departamento de Fisiologia, Universidade de São Paulo, São Paulo, Brazil; 5 Institute of Medical Physics and Biophysics, University of Leipzig, Leipzig, Germany; 6 Helmholtz Center for Infektion Research, Braunschweig, Germany; University of Lausanne, Switzerland

## Abstract

The protozoan parasite *Leishmania* is an intracellular pathogen infecting and replicating inside vertebrate host macrophages. A recent model suggests that promastigote and amastigote forms of the parasite mimic mammalian apoptotic cells by exposing phosphatidylserine (PS) at the cell surface to trigger their phagocytic uptake into host macrophages. PS presentation at the cell surface is typically analyzed using fluorescence-labeled annexin V. Here we show that *Leishmania* promastigotes can be stained by fluorescence-labeled annexin V upon permeabilization or miltefosine treatment. However, combined lipid analysis by thin-layer chromatography, mass spectrometry and ^31^P nuclear magnetic resonance (NMR) spectroscopy revealed that *Leishmania* promastigotes lack any detectable amount of PS. Instead, we identified several other phospholipid classes such phosphatidic acid, phosphatidylethanolamine; phosphatidylglycerol and phosphatidylinositol as candidate lipids enabling annexin V staining.

## Introduction

Lipids are essential for the structural and functional integrity of cells. As the predominant constituents of cellular membranes, lipids compartmentalize cellular functions and are involved in various aspects of signal transduction. One major class of lipids in eukaryotic cell membranes is represented by phospholipids, consisting of a glycerol backbone, two fatty acyl residues, and a polar head group at the *sn-3* position. The polar head group consists of a phosphate residue which is (except for phosphatidic acid, PA) esterified by an alcohol such as choline (to form phosphatidylcholine, PC) or ethanolamine (phosphatidylethanolamine, PE), the amino acid serine (phosphatidylserine, PS) or the carbohydrate inositol (phosphatidylinositol, PI). Among all these phospholipids, PS is a relatively minor constituent of most biological membranes. However, the low abundance of PS is outweighed by its physiological importance. Under normal conditions, PS is restricted to the inner plasma membrane leaflet in eukaryotic cells [1]. Any change in this distribution generally triggers a physiological event such as the clearance of apoptotic cells or the internalization of viruses by host cells [2], [3], [4].

PS has also been implicated in the infectivity of *Leishmania*, an obligate, intracellular parasite of humans and other mammals that infect cells of the mononuclear phagocyte lineage. The parasite has a digenic life cycle, residing as flagellated extracellular promastigote in the gut of the insect vector and as obligatory intracellular aflagellated amastigote found in the parasitophorous vacuoles of mammalian macrophages. A critical point in this host-parasite interaction involves the attachment to and invasion of host macrophages, initially by the promastigotes and subsequently by amastigotes. Both promastigotes and amastigotes use receptor-mediated phagocytosis for invasion. Furthermore, there is evidence that exposure of PS on the cell surface of the parasite mimics apoptosis and encourages the macrophages in the host organism to phagocytose the parasite [5], [6], [7], [8]. Notably, in these studies PS exposure has been detected by flow cytometry based on reactivity with either anti-PS antibodies or annexin V. However, despite being used extensively to detect externalization of PS, neither anti-PS antibodies nor annexin V are specific for this lipid and also bind other phospholipids such as phosphatidylglycerol (PG) and phosphatidylinositol-4,5-bisphosphate [9]. Thus, a direct proof for PS exposure by *Leishmania* parasites is currently lacking. Even the presence of PS in *Leishmania* has not been firmly established. Previous studies of *Leishmania* lipid compositions by thin layer chromatography-based methods have reported the presence of PS in several *Leishmania* species [10], [11], [12], while other studies based on mass spectrometry analysis failed to detect this lipid [13], [14], [15]. Here, we performed a combined analysis of *Leishmania* phospholipid classes and their ability to bind annexin V. Our findings show that upon permeabilization or miltefosine treatment *in vitro* grown *Leishmania* promastigotes are able to bind annexin V but lack any detectable amount of PS. Instead, we identified several other phospholipid classes as candidate lipids enabling annexin V staining.

## Results

### Annexin V Binding of *L. donovani* Promastigotes

To investigate whether *L. donovani* promastigotes can bind annexin V, we first permeabilized the parasites in the presence of 2.5 mM Ca^2+^ and 125 ng/mL annexin V-FITC by electroporation. This treatment resulted in strong FITC labeling of the parasites; in some cases, however, this labeling was restricted to inner structures (Supplementary Figure S1). By contrast, untreated parasites did not show a significant FITC labeling and, thus, binding of annexin V. Furthermore, we incubated parasites with miltefosine, a potent anti-leishmanial drug inducing an apoptosis like death [16], which resulted in annexin V-FITC and propidium iodide positive staining of the parasites (Supplementary Figure S1). Since annexin V preferentially interacts with membranes containing PS, we next analyzed total lipid extracts from untreated and miltefosin-treated *L. donovani* promastigotes for the presence of PS by thin-layer chromatography (TLC). Consistent with our previous results [17] we could neither detect significant concentrations of PS in the lipid extracts from untreated nor from miltefosine-treated parasites by this method.

### *L. donovani* Promastigotes Lack Phosphatidylserine

**Figure 1:**
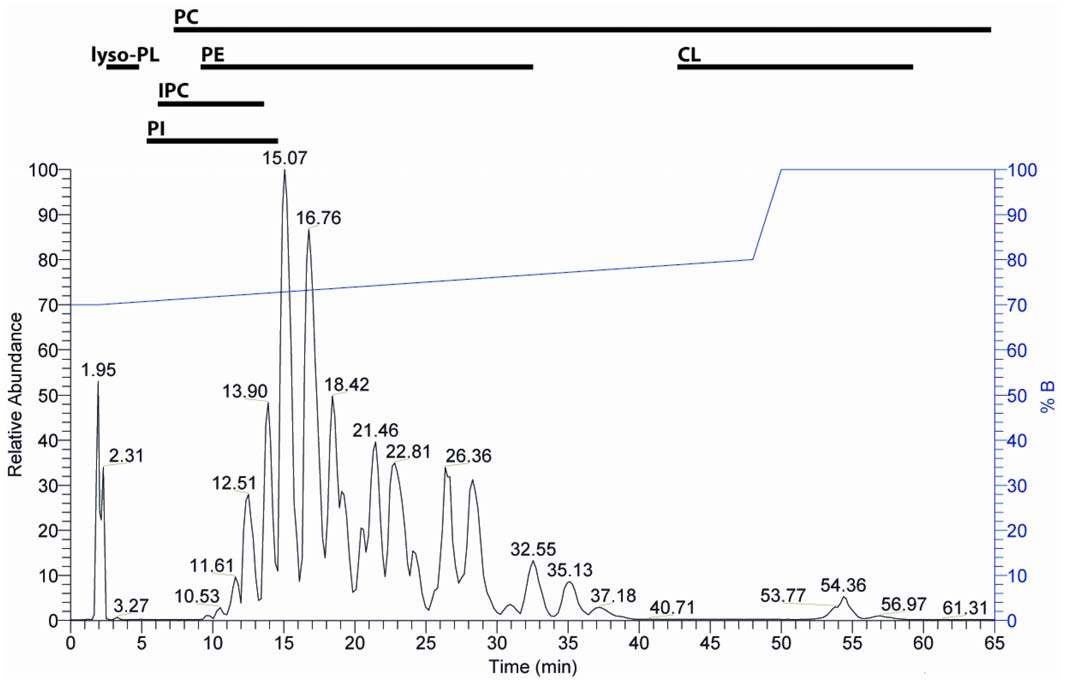
Base peak ion chromatogram of HPLC/MS analysis of a lipid extract of *L. donovani*. Lipids were separated using a BioBasic-4-column as described in Material and Methods. Elution was performed at a flow rate of 50 µl/min by increase of solvent B (70% acetonitrile, 25% 2-propanol, 5% water) vs. solvent A (95% water, 5% acetonitrile). The HPLC gradient is indicated at the right of the chromatogram. The intervals of the retention times of the individual lipid classes are labeled and the assignment of the peaks to the individual lipid species is available in **Tab. 1**. Abbreviations: CL, cardiolipin; IPC, inositolphosphorylceramide; lyso-PL, lyso-phospholipids; PC, phosphatidylcholine; PE, phosphatidylethanolamine; PI, phosphatidylinositol.

To corroborate that *L. donovani* promastigotes lack detectable levels of PS, total lipids were extracted from the parasites and fractionated by reversed phase HPLC coupled to electrospray ionization tandem MS using collision-induced dissociation. Major phospholipid species could be identified as: PC (mainly diacyl species), PE (diacyl and alkenylacyl (plasmalogen) species), PI (diacyl and alkenylacyl species), IPC and cardiolipins (Figure 1; Table 1). No hydrolysis of the plasmalogen species [18] due to the slight acidic conditions during the chromatographic separation was detected. We searched for PS species by scanning the MS^2^ spectra for the characteristic neutral loss of 87.0 amu (C_3_H_5_NO_2_) but no significant levels of PS could be detected by this highly sensitive MS method. To prove that low abundant PS species can be detected in biological extracts employing the described HPLC-MS method, 0.1% PS (18∶1/18∶1) was added to a phospholipid extract of *Escherichia coli*. In the HPLC-MS analysis of the spiked phospholipid extract of *E. coli* PS (18∶1/18∶1) was easily detectable even though it co-eluted with abundant PG species (Supplementary Figure S2). These PG species proved to give the highest ion yield and were thus eminently detectable in equimolar mixtures of six lipid standards containing PS (16∶0/18∶1), PE (16∶0/18∶1), PC (16∶0/18∶1), PG (16∶0/18∶1), PA (16∶0/18∶1) and cardiolipin (4×18∶1) (Figures S3, S4).

**Table 1:** Overview of the observed retention times in the HPLC chromatogram shown in Figure 1 and their assignment to the individual *L. donovani* phospholipid species.

Phospholipid^a^	Retention Time (min)^b^	Relative abundance (%)^c^
lyso-PE(18∶2)	2.9	Only this species detectable
lyso-PC(18∶3)	3.0	14.7
lyso-PC(18∶2)	3.3	49.6
lyso-PC(18∶1)	3.5	17.7
PC(18∶3/18∶2)	13.9	8.9
PC(18∶2/22∶6)	15.1	18.4
PC(18∶2/18∶2)	16.8	16.0
PC(18∶2/22∶5)	18.4	9.2
p-PC(16∶1a/18∶2)	23.3	33.3
p-PC(16∶0a/18∶2)	25.6	21.2
p-PC(18∶1a/18∶2)	32.6	21.8
p-PC(18∶0a/18∶2)	34.6	23.8
IPC(d16∶1/18∶0)	7.1	36.0
IPC(t16∶1/18∶0)	8.8	26.8
IPC(d18∶1/18∶0)	9.6	17.5
IPC(t18∶1/18∶0)	12.2	10.8
PE(2x18∶2)	13.4	25.1
PE(18∶2/18∶1)	17.4	21.5
PE(18∶0/18∶2)	23.0	16.1
PE(18∶0/18∶1)	28.7	11.7
p-PE(16∶1a/18∶2)	19.1	25.0
p-PE(16∶1a/18∶1)	24.1	15.7
p-PE(18∶1a/18∶2)	26.4	33.1
p-PE(18∶1a/18∶1)	32.6	13.5
PI(18∶1/16∶0)	10.8	20.8
PI(18∶2/18∶0)	11.7	16.0
PI(18∶1/18∶0)	14.6	59.3
p-PI(16∶0a/18∶1)	12.0	44.6
p-PI(18∶0a/18∶1)	17.2	55.4
CL(22∶6/18∶2, 22∶6/18∶2)	54.0	21.7
CL(22∶6/18∶2, 18∶2/22∶5)	54.4	39.0

a lyso-PE, lyso-phosphatidylethanolamine; lyso-PC, lyso-phosphatidylcholine; PC, phosphatidylcholine; p-PC, plasmalogen phosphatidylcholine; IPC, inositolphosphorylceramide; PE, phosphatidylethanolamine; p-PE, plasmalogen phosphatidylethanolamine; PI, phosphatidylinositol; p-PI, plasmalogen phosphatidylinositol; CL, cardiolipin. The letter “a” denotes an alkyl-or alkenyl-ether residue in the plasmalogen species. In all cases only the most abundant fatty acyl compositions are indicated, while the detailed evaluation of very minor species was not the subject of this paper. The inositolphosphorylceramides are denoted as follows: IPC (long chain base/fatty acyl residue) with the prefixes “d” and “t” to designate di-and trihydroxy species.

b Retention time of HPLC obtained from Figure 1.

c Percentage within the given phospholipid class as determined from the MS-signal intensities. The fatty acyl compositions were assigned to the *sn*-1 and 2 positions by means of the differing signal intensities in the fragment ion spectra.

### Phospholipid Analysis in *L. donovani* Promastigotes by MALDI–TOF Mass Spectrometry and ^31^P NMR Spectroscopy

**Figure 2:**
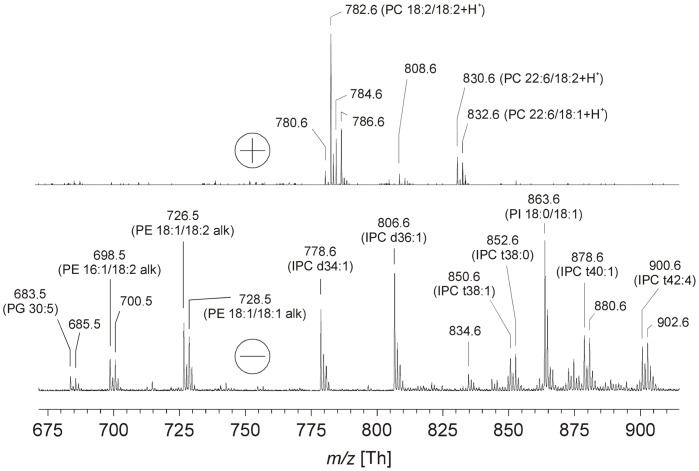
Lipid analysis by MALDI–TOF mass spectrometry. Positive and negative ion MALDI-TOF mass spectra of a total lipid extract of *L. donovani*. Peaks are marked with their *m/z* values and assignments are summarized in **Tab. 2**. DHB was used as the matrix for positive ion detection, while 9-AA was used in the negative ion mode. As the focus of this study was to clarify the potential presence of PS, only the mass range where PS could be expected is shown.

As an alternative analytic method to detect PS, total lipid extracts were subjected to matrix-assisted laser desorption and ionization time-of-flight (MALDI-TOF) mass spectrometry. This method is known to be affected only very moderately by sample impurities that might be present in the investigated extracts [19]. Only the mass regions where PS peaks could be expected are shown. The positive ion spectrum was found to contain mainly PC species (Figure 2, upper panel). This is not surprising because the permanent positive charge of the quaternary ammonium renders PC the highest detectability. This leads to the suppression of less sensitively detectable phospholipid species [20]. Such suppression effects are well known in the context of soft-ionization MS methods that generate “quasimolecular” ions. This is a particular problem regarding PE: as this phospholipid is zwitterionic, it is suppressed by PC in the positive ion mode and by more acidic phospholipids such as PS or PI in the negative ion mode [19]. In order to overcome potential suppression effects in the positive ion mode, an alkaline matrix (9-AA) that is more suitable than 2,5-dihydroxybenzoic acid (DHB) to record negative ion MALDI mass spectra [21] was also used. Negative ion MS detected PE, IPC and PI species (Figure 2, lower panel), while not even traces of PS were detectable. A detailed assignment of all detected peaks is provided in Table 2. No major efforts were undertaken to assign the fatty acyl compositions of IPC or to analyze cardiolipin that is rather difficult to detect by MALDI-TOF MS due to its higher mass in comparison to standard phospholipids [22], [23]. In addition to the analysis of the total extract, combined TLC/negative ion MALDI MS was also attempted in order to exclude effects of ion suppression: although PS was detectable in artificial lipid mixtures and after spiking the cellular extract with PS, no PS was detectable in the native extract. Thus, MALDI MS confirmed the ESI MS results (vide supra) and there is obviously no PS in *Leishmania* promastogotes.

Finally, lipid extracts were also analyzed by ^31^P NMR spectroscopy. This experimental approach provides the quantitative determination of all major phospholipid classes without the need of major sample work-up or separation into the individual lipid classes. The lipid mixture of interest is simply “solubilized” in a suitable detergent system in order to suppress the aggregation of phospholipids that would result in severe line-broadening and would make the differentiation of the individual phospholipid classes impossible [24]. However, we failed to detect any PS while the other phospholipid classes were easily detectable (Supplementary Figure S5). Although NMR suffers from much lower sensitivity in comparison to MS and should be cautiously interpreted, these NMR data are in line with the MS data (vide supra).

**Table 2:** Overview of the observed *m/z* values in the MALDI-TOF mass spectra shown in Figure 2 and their assignment to individual *L. donovani* phospholipid species.

m/z ratio	Polarity	Assignment^a^
683.5	−	PG 30∶5
685.5	−	PG 30∶4
698.5	−	PE 16∶1/18∶2 alk
700.5	−	PE 16∶0/18∶2 alk
726.5	−	PE 18∶1/18∶2 alk
728.5	−	PE 18∶1/18∶1 alk
778.6	−	IPC d34∶1
780.6	+	PC 18∶2/18∶3+ H^+^
782.6	+	PC 18∶2/18∶2+ H^+^
784.6	+	PC 18∶1/18∶2+ H^+^ or PC 18∶3/18∶0+ H^+^
786.6	+	PC 18∶2/18∶0+ H^+^
806.6	−	IPC d36∶1
808.6	+	PC 22∶5/16∶0+ H^+^
830.6	+	PC 22∶6/18∶2+ H^+^
832.6	+	PC 22∶6/18∶1+ H^+^
834.6	−	IPC d38∶1
850.6	−	IPC t38∶1
852.6	−	IPC t38∶0
863.6	−	PI 18∶0/18∶1
878.6	−	IPC t40∶1
880.6	−	IPC t40∶0
900.6	−	IPC t42∶4
902.7	−	IPC t42∶3

a In the case of acidic phospholipids (negative ion spectra), the sodium salt was considered as the neutral molecule. See “Material and Methods” section for further details.

### Lack of PS is a General Feature of *Leishmania* Promastigotes

**Figure 3:**
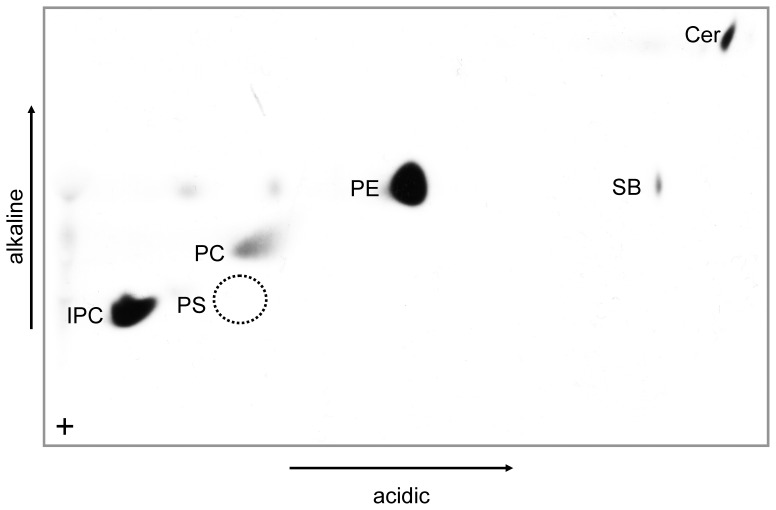
Analysis of [3-^3^H]serine-labeled lipids from *L. donovani* parasites. Promastigotes were labelled for 16 h with [3-^3^H]serine. Lipids from 10^8^ parasites were extracted, separated by two-dimensional thin layer chromatography, and then visualized by fluorography. The location of individual lipid species was verified by ESI and MALDI-TOF mass spectrometry. Unidentified lipids are not marked. Cer, ceramide, PC, phosphatidylcholine; PE, phosphatidylethanolamine; IPC, inositolphosphorylceramide; SB, sphingosine. The position of the phosphatidylserine standard (PS) is enclosed by a dotted circle.

To investigate whether *Leishmania* parasites lack PS in general, total lipid extracts were prepared from *L. major, L. mexicana, L. amazonensis, L. tropica, L. guyanensis and L. shawi* promastigotes and analyzed by TLC-MALDI imaging [25]. None of the investigated parasite strains showed any detectable amounts of PS (data not shown), although it is normally detectable in amounts of about 30 pg [26]. Further analysis of the lipid extracts by HPLC coupled electrospray ionization tandem MS did also not reveal the presence of PS in any of the tested strains (Supplementary Table S1 and data not shown). Finally, we metabolically labeled the various parasite species with [^3^H]serine and subsequently analyzed for its incorporation into total lipids by TLC. In all parasite species, serine was abundantly incorporated into both glycerophospholipids (PE and PC) and sphingolipids (ceramides and IPC) but no radiolabel signal was obtain for PS (Figure 3 and data not shown). We conclude that *Leishmania* promastigotes do not synthesize PS.

### Identification of Annexin V Binding Lipid Species in *L. donovani* Promastigotes

**Figure 4:**
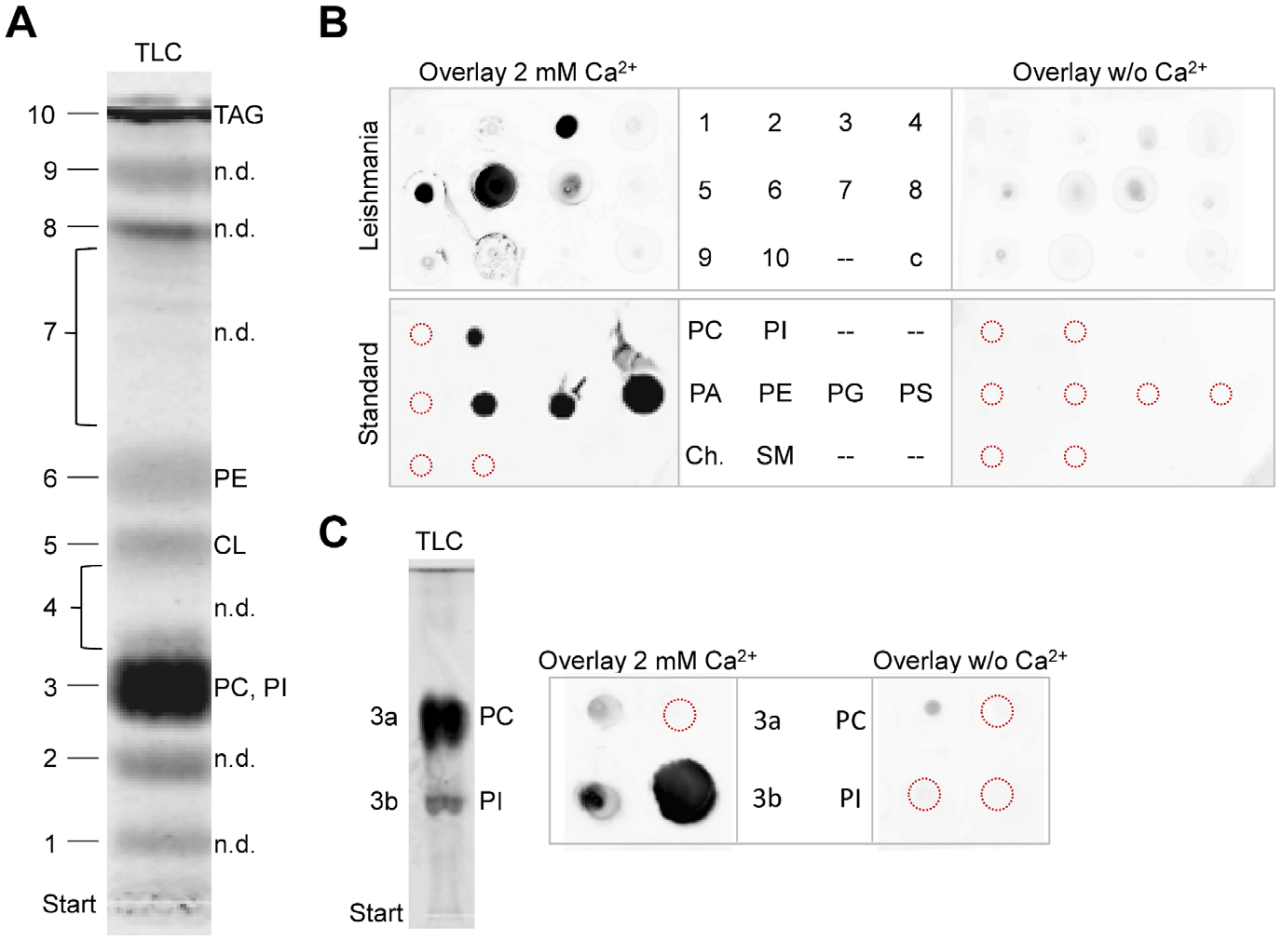
Overview of various lipids from *L. donovani* and their annexin V-binding ability. (A) A total lipid extract from *L. donovani* promastigotes was separated by one-dimensional TLC using chloroform/methanol/water (65/25/4, v/v/v) as described in “Material and Methods”. The individual lipid species were visualized by primuline staining, scraped off, extracted and subjected to MALDI-TOF and ESI mass spectrometry. Chromatograms shown are scanned by a Typhoon imager as described in Material and Methods. Regions (1–10) are marked and assignments are indicated. Abbreviations used in assignments: PA, phosphatidic acid; PC, phosphatidylcholine; PE, phosphatidylethanolamine; PG, phosphatidylglycerol; PI, phosphatidylinositol; PS, phosphatidylserine; TAG, triacylglycerols; CL, cardiolipin, n.d., not determined. (B) Overlay assays. *Leishmania*: Individual lipids extracted from TLC region 1–10 were spotted onto nitrocellulose and incubated with annexin V-FITC in the presence and absence of Ca^2+^; an unloaded TLC region scraped off and treated exactly as regions 1–10 served as background control for the primuline signal (marked c). *Standard*: PC (18∶1/18∶1), PI, PA (18∶1/18∶1), PE (18∶1/18∶1), PG (18∶1/18∶1), PS (18∶1/18∶1), stearoyl-sphingomyelin (SM 18∶0), and cholesterol (Ch.) served as controls and emphasize the specificity of the assay. Location of spotted lipids is indicated with red broken circles. (C) Lipids extracted from TLC region 3 were subjected to one-dimensional TLC using chloroform/methanol/25% aqueous ammonium hydroxide (90/54/7, v/v/v) and stained with primuline. PC and PI were identified by MALDI-TOF mass spectrometry. Lipids from regions 3a and 3b were eluted, re-protonated and used for overlay assay with annexin V-FITC in the presence and absence of Ca^2+^.

To identify *Leishmania* lipids that bind annexin V, lipids were extracted from *L. donovani* promastigotes and separated by TLC using chloroform/methanol/water (65/25/4, v/v/v) as solvent system (Figure 4A). From ten individual TLC spots, lipids were extracted and analyzed by overlay assays [27]. To this end, lipids were spotted onto nitrocellulose and incubated with annexin V-FITC in the presence and absence of Ca^2+^. Under these conditions, *Leishmania* lipids extracted from regions 3 (PC, PI), 5 (cardiolipin), and 6 (PE) were found to bind to annexin V-FITC when Ca^2+^ was present (Figure 4B). Next, lipids from TLC region 3 were subjected to a second TLC separation using an alkaline solvent system to separate PI and PC (Figure 4C). Upon extraction and re-protonation, both *Leishmania* lipids were examined for binding by annexin V-FITC in overlay assays. Ca^2+^-dependent binding was detected for PI but not for PC lipids (Figure 4C). Control experiments with several standard lipids showed binding of annexin V-FITC to PS, PG, PE, and PI in the presence of Ca^2+^, while binding to PC, PA, SM, and cholesterol was negligible under these conditions (Figure 4B).

**Figure 5:**
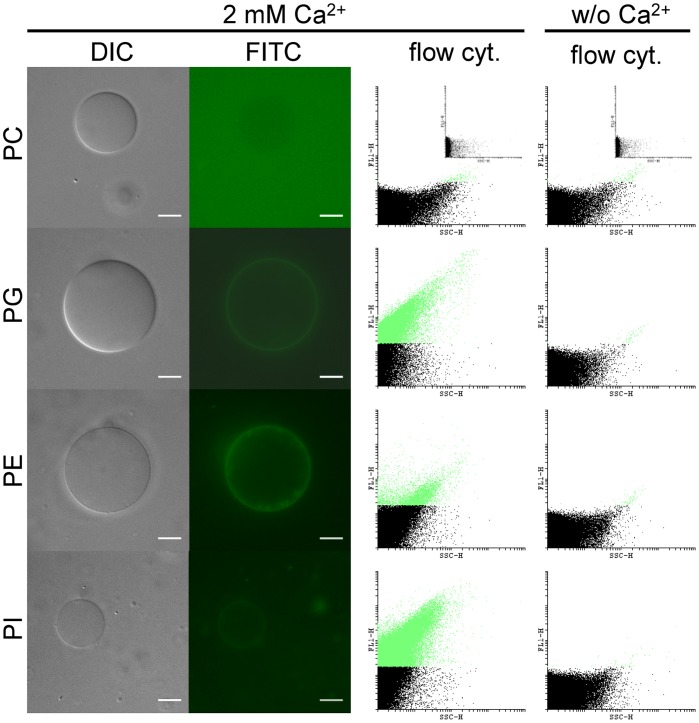
Binding of annexin V-FITC to giant vesicles. Giant unilamelar vesicles were prepared from different lipids and incubated with annexin V-FITC in the presence or absence of 2 mM Ca^2+^. Vesicles were analyzed by differential interference contrast (DIC) and fluorescence microscopy (FITC), or by flow cytometry (flow cyt.). Dot plots show side scattering (SSC-H) and fluorescence (FL1-H) on non-gated samples. Vesicles exceeding the fluorescence signal of buffer alone appear green colored. *Insets* correspond to buffer (with annexin V-FITC) without vesicles in the presence and absence of calcium ions. PC: PC (18∶1/18∶1) only; PG: PC (18∶1/18∶1)/PG (18∶1/18∶1), (9/1, mol/mol); PE: PC (18∶1/18∶1)/PE (18∶1/18∶1), (9/1, mol/mol); PI: PC (18∶1/18∶1)/PI, (9/1, mol/mol). Bar, 10 µm.

To test whether the individual lipid classes identified in the overlay assays also promote annexin V-FITC binding when incorporated in lipid membranes, giant vesicles of different lipid compositions were generated and incubated with annexin V-FITC in the presence or absence of Ca^2+^ ions. Fluorescence microscopy revealed Ca^2+^-dependent binding of annexin V-FITC to membranes containing PG, PE, or PI but not to pure PC vesicles (Figure 5; Supplementary Figure S6). Annexin V conjugated with Alexa 568 as fluorophore gave equivalent results (Supplementary Figure S7). Quantitative assessment of annexin V-FITC binding to giant vesicles by flow cytometry revealed the strongest labelling for vesicles containing PG and PI. Labelling of vesicles containing PE was moderate while pure PC vesicles hardly exhibited fluorescence above the background contribution of the buffer (insets in uppermost flow cytometry graphs). Collectively, these data show that binding of annexin V is not restricted to PS but includes several other phospholipids such as PE, and particularly the acidic PI and cardiolipin that are present in *Leishmania* parasites in considerable amounts.

## Discussion

In this study, we performed a combined lipid analysis of *Leishmania* promastigotes using three different techniques: thin-layer chromatography, (ESI and MALDI) MS and phosphorus-31 nuclear magnetic resonance spectroscopy. Based on these three complementary approaches, PC, PE, PI, PG, cardiolipin and inositol phosphorylceramide phospholipids were clearly detectable but there was no evidence for the presence of PS in lipid extracts derived from several *in vitro* grown *Leishmania* species, suggesting that the parasite does not synthesize PS under these conditions. Consistent with this notion, ^3^H-serine labelling experiments further validated the absence of PS in promastigotes of several *in vitro* grown *Leishmania* species.

These findings are in line with several other MS analyses that found no evidence for the presence of PS in lipid extracts of *L. donovani* [15] and *L. major* promastigotes [13], [14] but contrast with a recent MS study reporting the presence of PS in *L. donovani* promastigotes [28]. A potential reason for this discrepancy could be that in certain growth states/conditions such as late logarithmic phase, the parasites do synthesize PS (see below). The parasites may also be able to acquire PS from foetal calf serum that contains several phospholipids including PS [29] and is commonly used as a supplement to the culture media.

Beside *L. donovani* and *L. major,* promastigotes of *L. mexicana, L. amazonensis, L. tropica, L. guyanensis,* and *L. shawi* did not contain any detectable levels of PS neither in TLC-MALDI “imaging” nor in HPLC coupled electrospray ionization tandem mass spectrometry. Nevertheless, several of these parasite species have been reported to bind annexin V under certain conditions [16], [30], [31], [32]. In the present report, we observed strong staining of *L. donovani* promastigotes by annexin V upon electroporation or miltefosine treatment despite the absence of detectable levels of PS. These observations call for caution regarding the use of fluorescence-labeled annexin V as the only approach to probe the presence of PS because our results imply that the parasites expose other lipids than PS that enable annexin V binding.

Using lipid overlay assays and giant vesicle membranes we found that annexin V binds not only to PS but also to several other phospholipids including PE and PI. In addition, annexin V has been reported to bind PA and phosphatidylinositol-4,5-bisphosphate [9]. Similar to PS, all these phospholipid species are enriched to varying extents on the cytoplasmic side of eukaryotic membranes, while sphingolipids (i.e. sphingomyelin and glycosphingolipids) are enriched in the exoplasmic leaflet [33]. During apoptosis, however, this asymmetric lipid arrangement in the plasma membrane is lost, resulting in drastic changes in the phospholipid composition of both leaflets [34], [35], [36], [37]. Binding of annexin V to the cell surface of *Leishmania* parasites is therefore likely to be a consequence of changes in the plasma membrane lipid arrangement.

The lack of significant amounts of PS in *Leishmania* promastigotes implies that the parasite cannot take advantage of surface exposed PS during the initial infection process and calls into question the concept of PS-based apoptotic mimicry. In fact, this concept has not been firmly established for *Leishmania* parasites and is essentially based on the labeling of parasite (sub)populations with either anti-PS antibodies or annexin V. Our data suggest that the parasite may rearrange the plasma membrane distribution of other phospholipids such as PI, PE, PG and/or PA. Resting red cells, however, do not bind Annexin V in the presence of low calcium concentrations (i.e. 1 to 2 mM) although their outer membrane leaflet contains ∼20% of PE (accounting for ∼11 mol% of all phospholipids in this monolayer) and ∼20% of PI (accounting for ∼1 mol% of all phospholipids in this monolayer) [38]. Plasma membranes of *L. donovani* were found to contain an amount of PE, PI and PG that represented about 35, 4 and 2% of the total phospholipids [39]. Taken into account that the extent to which annexin V binds to a membrane is a complex function related to the membrane lipid composition, the free annexin V concentration and the calcium concentration [40], it is likely that global changes in the transbilayer arrangement of PE, PI, PG and/or PA trigger annexin V staining of the parasites. In support of this notion, we recently found that parasites lacking a plasma membrane lipid transporter but not wild-type parasites are strongly labeled by PE-binding peptides [17]. It remains to be evaluated whether PE, PI, PG and/or PA do contribute to the infectivity of parasites.

The genomes of *Leishmania* and other trypanosomatids encode two genes showing similarity to eukaryotic phosphatidyl serine/ethanolamine base exchange enzymes [14]. This base exchange/decarboxylase cycle could in principle function to provide the parasites with PE rather than PS, thus accounting for its presence in the genome in the absence of detectable amounts of PS. However, studies by Zhang et al. [14], [41], [42] revealed that this pathway does not contribute to PE synthesis in *Leishmania* promastigotes. Instead, recent reports indicate that trypanosomes do synthesize PS via this pathway by head group exchange with PE [43], [44], [45]. Given that all experiments undertaken in the present work were performed on the promastigote stage of the parasite, it might be possible that *Leishmania* amastigotes synthesize this lipid and regulate its transbilayer distribution in the parasite plasma membrane. Furthermore, loss of plasma membrane lipid asymmetry as a mechanism for survival in the host has been described for several protozoan parasites [46]. The analytical procedures described here should be useful for defining more precisely the phospholipid types exposed on their cell surfaces and help to uncover their potential role in parasite infectivity.

## Materials and Methods

### Materials

Annexin V-FITC and Annexin V-Alexa 569 were purchased from VPS-Diagnostics (Hoeven, The Netherlands) and Roche Diagnostics (Mannheim, Germany), respectively. PI was purchased as ammonium salt solution in chloroform from Sigma-Aldrich (Taufkirchen, Germany); all other lipids were obtained from Avanti Polar Lipids (Birmingham, AL, USA). High performance TLC silica gel 60 plates were from Merck (Darmstadt, Germany). Lipids were used without further purification. All solvents used were of the purest grade available. Unless indicated otherwise, all other chemicals and reagents were obtained from Sigma-Aldrich (Taufkirchen, Germany) and used as supplied.

### Parasites

Promastigotes of *L. donovani* (MHOM/ET/67/HU3, kindly provided by Francisco Gamarro, Instituto de Parasitología y Biomedicina, Granada, Spain; MHOM/IN/80/Dd8) were grown at 26°C in M-199 medium (Invitrogen, Karlsruhe, Germany) supplemented with 40 mM HEPES, 100 µM adenosine, 500 ng/mL hemin, 10 µM 6-biopterin and 10% heat-inactivated fetal calf serum (Gibco, Invitrogen GmbH, Karlsruhe, Germany). Promastigotes of *L. major* (MHOM/IL/81/Friedlin), *L. mexicana* (MNYC/BZ/1962/M379), *L. amazonensis* (MHOM/BR/1973/M2269*), L. tropica* (M6662), and *L. guyanensis* (MHOM/BR/1975/M4147) and *L. shawi* (MCEB/BR/1984/M8408) were grown at 25°C in M199 (Invitrogen, Grand Island, NY, USA) supplemented with 10% fetal bovine serum (Invitrogen) and 2% human urine. For metabolic labeling, promastigotes (10^7^ cells/ml) were labeled with 10 µCi/ml [3-^3^H]serine (specific activity 30 Ci/mmol) for 16 h at 26°C.

### Preparation of Giant Vesicles

Giant unilamellar vesicles were produced from lipid films dried on indium tin oxide (ITO) coated glass slides by electroswelling as originally described by Angelova *et al*. [47]. Shortly, lipid mixtures were made from 5 mM stock solutions in chloroform kept at −20°C. Lipids were mixed in a final volume of 50 µL chloroform (2 mM final total lipid concentration). This solution was dried under a gentle stream of N_2_ to form a lipid film and then re-solubilized in 80 µL chloroform. Single droplets of this lipid mixture were spread onto two ITO coated glass slides (Präzisions Glas & Optik GmbH, Iserlohn, Germany). To obtain homogeneously distributed lipid films, the solvent was evaporated on a heater plate at 50–60°C. To get rid of traces of the solvent glass slides were placed under vacuum (<40 mbar) for 1 h. The electroswelling chamber was assembled from both lipid ITO coated slides using 1 mm teflon spacers. The chamber was filled with 1.5 ml of sucrose-buffer (250 mM sucrose; 7.5 mM NaN_3_). An alternating voltage (rising from 0.02 V to 1.1 V over 30 min) with a frequency of 4 Hz was applied and the frequency was raised to 10 Hz in 30 s. Vesicles were formed during 3 h incubation. To detach the vesicles, a voltage of 1.3 V (4 Hz) was applied for 30 min. The vesicles were stored at room temperature for up to 5 days. For microscopy, the vesicle solution was diluted 1∶2 with binding buffer (150 mM NaCl, 5 mM KCl, 1 mM MgCl_2_, 10 mM HEPES, pH 7.4) with addition of either 2 mM MgCl_2_ (Ca^2+^-free binding buffer) or 2 mM CaCl_2_ (Ca^2+^-containing binding buffer)_._

### *In vitro* Lipid Binding Assay with Annexin V-FITC

For protein lipid overlay assays, nitrocellulose membranes were first spotted with various indicated lipid species (500 pmol) dissolved in chloroform and blocked in binding buffer supplemented with 2% (w/v) fatty acid-free BSA for 30–60 min. After blocking, membranes were incubated for 15 min in the darkness with annexin V-FITC (250 ng/mL) in Ca^2+^-free or Ca^2+^-containing binding buffer. Thereafter, blots were washed three times in the respective binding buffer and scanned for FITC fluorescence using a Typhoon Trio variable-mode imager (GE Healthcare, Uppsala, Sweden) equipped with a 488 nm argon laser and a 526 nm short pass filter. To study annexin-V binding to lipid membranes, giant vesicles were diluted 1∶2 with binding buffer supplemented with 0.2% (w/v) fatty acid-free BSA and 250 ng/mL annexin V-FITC. After 10 min incubation in the darkness, GUVs were either observed in differential interference contrast (DIC) or in fluorescence mode (excitation filter BP 470/40, beam splitter 500, band-pass filter 525/50) using an inverted fluorescence microscope (Leica DM 4000 B, Wetzlar, Germany). Images were acquired with a black-and-white camera (Leica DFC340 FX). Fluorescent images were coloured using the green LUT settings of the program LAS AF Lite (Leica). Flow cytometry analysis was performed on a FACS Calibur instrument (BD Biosciences, San Jose, CA) equipped with an argon laser (488 nm) using Cell Quest software. Fluorescence was detected through a 530/30 band-pass filter. For each sample, data from 10,000 events were collected without gating. All experiments were performed at ambient conditions.

### Lipid Extraction and Thin-layer Chromatography

Log phase promastigotes (10^8^ cells) were harvested by centrifugation (1000×g, 10 min), washed twice with PBS and suspended in PBS. Total cellular lipids were extracted by the method of Bligh and Dyer [48] and applied on TLC either manually or by means of the sample applicator Linomat IV (Camag, Muttenz, Switzerland). For one-dimensional TLC, plates were developed either in chloroform/ethanol/water/triethylamine (30/35/7/35, v/v/v/v), in chloroform/methanol/water (65/25/4, v/v/v), or chloroform/methanol/25% aqueous ammonium hydroxide (90/54/7, v/v/v). For two-dimensional thin-layer chromatography, plates were first developed in chloroform/methanol/25% aqueous ammonium hydroxide (90/54/7, v/v/v) followed by chloroform/acetone/methanol/acetic acid/water (50/20/10/10/5, v/v/v/v/v) for the second dimension. Lipids and standards were visualized with common lipid-locating agents such as iodine, ninhydrin (0.25% ninhydrin w/v in acetone), or primuline (5 mg primuline in 100 ml aceton/water, 80/20, v/v). The spots were assessed using a digital image system in combination with the program Argus X1 (BioStep, Jahnsdorf, Germany) and a Typhoon Trio variable-mode imager (GE Healthcare). For further analysis, lipid spots from primuline stained 1D-TLC or iodine stained 2D-TCL were scraped off and extracted as described for parasites. Lipids (extracted from TLC plates after separation under alkaline conditions) had to be protonated to regain binding affinity of annexin V-FITC. For this, recovered lipids were solubilized in chloroform/methanol (2∶1, v/v) at a concentration of about 0.4 mg/ml. Next, one fifth of the solutiońs volume was added as water to obtain an aqueous layer and to enable phase separation. Thereafter, 1 µmol HCl was added and the solution was mixed. After centrifugation (500×g, 1 min), the chloroform phase was collected, dried and used for lipid protein overlay assays [27]. The ^3^H-containing radioactive lipids were detected by fluorography; TLC plates were dipped in 0.4% 2,5-diphenyloxazol dissolved in 2-methylnaphthalene supplemented with 10% xylene and exposed to Kodak X-Omat S films at-80°C. Total phospholipid content was determined by digesting lipid samples in deionized water and perchloric acid for 1 h at 180°C followed by addition of ammonium molybdate and ascorbic acid. After further heating for 5 min in a boiling water bath, the sample was cooled and the absorbance was read at 797 nm to quantify total lipid phosphorus.

### HPLC Coupled to Electrospray Ionization Tandem Mass Spectrometry

High performance liquid chromatography (HPLC) separation of the total lipid extracts was carried out on an Agilent 1200 system (Agilent) equipped with a BioBasic-4 column (C4, 150 mm×1 mm i.d., particle size 5 µm, ThermoFisher Scientific). A Finnigan LTQ Fourier transform ion cyclotron resonance (FTICR) mass spectrometer (ThermoFisher Scientific) equipped with a 6 Tesla superconducting magnet was used for subsequent mass analysis. Lipid species were assigned according to their accurate masses in negative ionization mode and their characteristic fragmentation patterns. PCs were detected as acetate adducts [M+AcO]^−^, whereas the other PLs were detected as deprotonated molecules [M-H]^−^. In the fragmentation (MS/MS) experiments, PCs and PSs showed characteristic neutral losses: PCs simultaneously lost the acetate counter ion and one *N-methyl* group (C_3_H_6_O_2_, 74.0 amu), PSs lost the head group (C_3_H_5_NO_2_, 87.0 amu) as commonly observed in fragmentation experiments with these negative ions [49].

The fatty acyl residues of all glycerophospholipids were identified as [FA-H]^−^ ions either in MS^2^ or in MS^3^ experiments. The fatty acyl residues of inositolphosphorylceramides (IPCs) were detected as neutral losses in MS^2^ or in MS^3^ experiments fragmenting [M-HG-H]^−^ ions [50]. The mass spectrometer was calibrated according to the manufacturer’s recommendations and transfer optics were tuned with a lipid standard mixture containing PS (16∶0/18∶1), PG (16∶0/18∶1), PA (16∶0/18∶1), PE (16∶0/18∶1), PC (16∶0/18∶1) and cardiolipin (4×18∶1). The following parameters were used for fragmentation experiments using collision-induced dissociation: normalized collision energy: 30–40%, activation: q = 0.25, activation time: 30 ms. Extracted lipids (about 0.5 mg) were re-solubilized in 200 µl acetonitrile/methanol (1∶1, v/v) and 4 µl were injected. The employed HPLC-MS method was adopted from Hein *et al.* [51]. Eluent A consisted of 95% water and 5% acetonitrile (v/v), eluent B of 70% acetonitrile, 25% 2-propanol and 5% water (v/v). Both eluents contained 10 mM triethylammonium acetate and 1 mM acetic acid. The gradient elution used was as follows: 0–2 min 70% B, 2–48 min 70%–80% B, 48–50 min 80%–100% B, 50–73 min 100% B, 73–75 min 100%–70% B, 75–90 min 70% B at a flow rate of 50 µl/min and 40°C column temperature. The mass spectrometric data were processed and evaluated using the Profiler-Merger-Viewer software tool [52].

### MALDI–TOF Mass Spectrometry

For the acquisition of the positive or negative ion mass spectra, 0.5 mol/L (ca. 77 mg/mL) 2,5-dihydroxybenzoic acid solution in methanol) or 10 mg/mL 9-aminoacridine (9-AA; in isopropanol/acetonitrile, 60/40, v/v) were used, respectively [53], [54]. As the quality of the spectra recorded in the presence of 9-AA depends significantly on the applied solvent system, the applied lipid extracts were diluted with isopropanol/acetonitrile (60/40, v/v). All samples were pre-mixed with the matrix prior to deposition onto the MALDI target. All MALDI-TOF mass spectra were acquired on a Bruker Autoflex mass spectrometer (Bruker Daltonics, Bremen, Germany). The system utilizes a pulsed nitrogen laser, emitting at 337 nm. The extraction voltage was 20 kV and gated matrix suppression was applied to prevent the saturation of the detector by matrix ions [55]. For each mass spectrum, 128 single laser shots were averaged. The laser fluence was kept about 10% above threshold to obtain optimum signal-to-noise (S/N) ratios. In order to enhance the spectral resolution all spectra were acquired in the reflector mode using delayed extraction conditions. Assignments of fatty acyl residues to *sn*-1 and *sn*-2 positions are based on subsequent region-specific digestion with the enzyme phospholipase A_2_. More detailed methodological descriptions of MALDI-TOF-MS are available in Fuchs *et al.* [19].

## Supporting Information

Figure S1 Annexin V-binding of *L. donovani* promastigotes. Early log-phase parasites (10^6^ cells/mL) were cultured in the absence (control) or the presence of 40 µM miltefosine (hexadecylphosphocholine, Calbiochem, La Jolla, CA) for 10 h, washed and suspended at a concentration of 10^6^ parasites/ml in annexin V-binding buffer (140 mM NaCl, 2.5 mM CaCl_2_, 10 mM HEPES, pH 7.4). Aliquots (0.5 ml) of this suspension were incubated on ice for 10 min in the presence of 125 ng annexin V-FITC and 1 µg propidium iodide (ProI). For electroporation, two electric pulses (160 ms, 1100 V) produced by an Eppendorf Multiporator were applied to the cell suspension. Subsequently, the samples were analyzed by differential interference contrast (DIC) and fluorescence (ProI, FITC) by confocal laser scanning microscopy (FluoView 1000, Olympus, Tokio, Japan) using a 60× (numerical aperture 1.35) oil-immersion objective. Fluorescence of FITC was excited with a 488 nm argon laser and recorded between 500 and 530 nm. Fluorescence of ProI was excited with a 559 nm argon laser and recorded between 570 and 600 nm. Images with a frame size of 256×256 pixels were acquired. Bar, 10 µm.
(TIF)

Figure S2 Ion chromatogram of HPLC/MS analysis of a phospholipid extract of *Escherichia coli* supplemented with 0.1% PS (18∶1/18∶1). Lipids were separated using a BioBasic-4-column as described in “Materials and Methods”. Elution was performed at a flow rate of 50 µL/min by increase of solvent B (70% acetonitrile, 25% 2-propanol, 5% water) vs. solvent A (95% water, 5% acetonitrile). Shown in blue is the trace for the intensity of PS which co-elutes with PG (highlighted by a red bar in the chromatogram). The intervals of the retention times of the individual lipid classes are labeled at the top of the chromatogram. Abbreviations: CL, cardiolipin; PE, phosphatidylethanolamine; PG, phosphatidylglycerol; PS, phosphatidylserine. *Inset*: Negative ESI-FTICR mass spectrum recorded during the elution of PG classes and PS (18∶1/18∶1).
(TIF)

Figure S3 Ion chromatogram of HPLC/MS analysis of an equimolar mixture of the lipid standards PS (16∶0/18∶1), PE (16∶0/18∶1), PC (16∶0/18∶1), PG (16∶0/18∶1), PA (16∶0/18∶1) and CL (4×18∶1). All lipids were used in a concentration of 10 µM in acetonitrile/water/2-propanol/methanol (44.6/36.9/13.5/5, v/v/v/v) and 4 µl were injected into the MS device. Lipids were separated using a BioBasic-4-column as described in “Materials and Methods”. Elution was performed at a flow rate of 50 µL/min by increase of solvent B (70% acetonitrile, 25% 2-propanol, 5% water) vs. solvent A (95% water, 5% acetonitrile).
(TIF)

Figure S4 Negative ESI-FTICR mass spectrum of an equimolar mixture of the lipid standards PS (16∶0/18∶1), PE (16∶0/18∶1), PC (16∶0/18∶1), PG (16∶0/18∶1), PA (16∶0/18∶1) and CL (4×18∶1) injected directly. All lipids had a concentration of 1 µM in acetonitrile/water (7/3, v/v). 0.3% triethylamonium acetate was used to enhance ion generation. PC is detected as acetate adduct. *Inset*: amplified region with the PE species.
(TIF)

Figure S5 ^31^P NMR spectra of *L. donovani*. Parasites were washed and re-solubilized in 200 mM sodium cholate, 5 mM EDTA in D_2_O. ^31^P NMR spectra were recorded on a Bruker DRX-600 spectrometer operating at 242.94 MHz. All measurements were performed on 0.6 ml samples in 5 mm NMR tubes using a 5 mm “direct” broadband probe at 37°C. Composite pulse decoupling (Waltz-16) was applied to eliminate ^31^P-^1^H coupling. Other NMR parameters were as follows: Data size: 16 k, 60° pulse (5 µs), pulse delay 2 s. A line broadening of 2 Hz was applied for the processing of the free induction decays. Chemical shift assignments were externally referenced relative to 85% orthophosphoric acid at 0.00 ppm. Abbreviations used in peak assignments: PC, phosphatidylcholine; PE, phosphatidylethanolamine; PI, phosphatidylinositol; Pi, inorganic phosphate.
(TIF)

Figure S6 Giant unilamelar vesicles were prepared from different lipids and incubated with annexin V-FITC in the absence of Ca^2+^ (w/o Ca^2+^). Vesicles were analyzed by differential interference contrast (DIC) and fluorescence microscopy (FITC). PC: PC (18∶1/18∶1) only; PG: PC (18∶1/18∶1)/PG (18∶1/18∶1), (9/1, mol/mol); PE: PC (18∶1/18∶1)/PE (18∶1/18∶1), (9/1, mol/mol); PI: PC (18∶1/18∶1)/PI, (9/1, mol/mol). Bar, 10 µm.
(TIF)

Figure S7 Giant unilamelar vesicles were prepared from different lipids and incubated with Annexin V-Alexa 568 (2 µL/mL; Roche Diagnostics, Mannheim, Germany) in the presence or absence of Ca^2+^. Vesicles were analyzed by differential interference contrast (DIC) and fluorescence microscopy (Alexa; excitation band-pass filter 515–560, beam splitter 580, emission long-pass filter 590). PC: PC (18∶1/18∶1) only; PG: PC (18∶1/18∶1)/PG (18∶1/18∶1), (9/1, mol/mol); PE: PC (18∶1/18∶1)/PE (18∶1/18∶1), (9/1, mol/mol); PI: PC (18∶1/18∶1)/PI, (9/1, mol/mol). Bar, 10 µm.
(TIF)

Table S1 HPLC/MS analysis of a lipid extract of L. amazonensis.
(DOC)
