# Supplementary figures and images for: Leishmania Promastigotes Lack Phosphatidylserine but Bind Annexin V upon Permeabilization or Miltefosine Treatment
Source: PLoS One. 2012 Aug 1;7(8):e42070. doi: 10.1371/journal.pone.0042070 (PMC3411662; doi:10.1371/journal.pone.0042070)

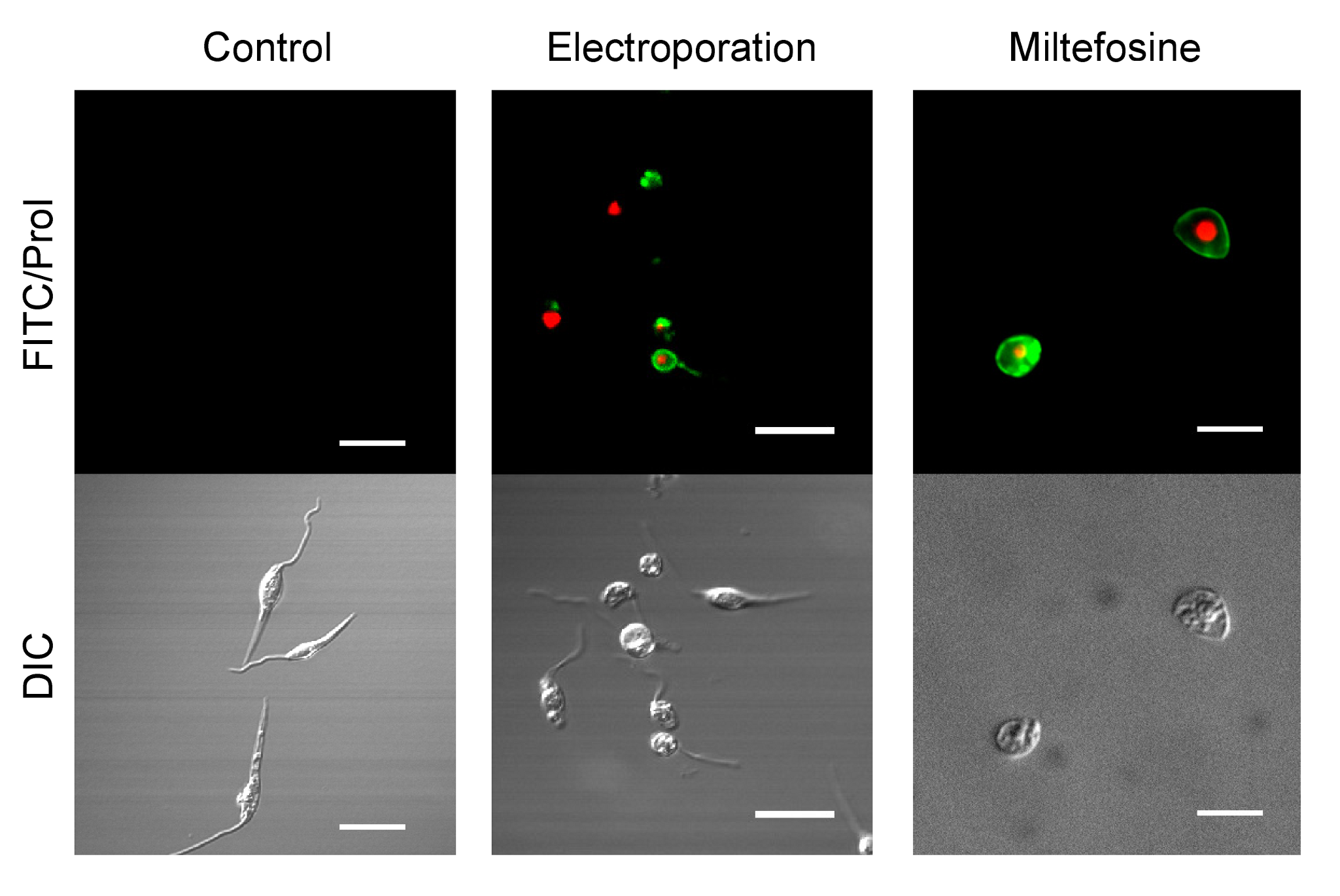

Supplement: Figure S1 — Annexin V-binding of L. donovani promastigotes. Early log-phase parasites (106 cells/mL) were cultured in the absence (control) or the presence of 40 µM miltefosine (hexadecylphosphocholine, Calbiochem, La Jolla, CA) for 10 h, washed and suspended at a concentration of 106 parasites/ml in annexin V-binding buffer (140 mM NaCl, 2.5 mM CaCl2, 10 mM HEPES, pH 7.4). Aliquots (0.5 ml) of this suspension were incubated on ice for 10 min in the presence of 125 ng annexin V-FITC and 1 µg propidium iodide (ProI). For electroporation, two electric pulses (160 ms, 1100 V) produced by an Eppendorf Multiporator were applied to the cell suspension. Subsequently, the samples were analyzed by differential interference contrast (DIC) and fluorescence (ProI, FITC) by confocal laser scanning microscopy (FluoView 1000, Olympus, Tokio, Japan) using a 60× (numerical aperture 1.35) oil-immersion objective. Fluorescence of FITC was excited with a 488 nm argon laser and recorded between 500 and 530 nm. Fluorescence of ProI was excited with a 559 nm argon laser and recorded between 570 and 600 nm. Images with a frame size of 256×256 pixels were acquired. Bar, 10 µm. (TIF) [file pone.0042070.s001.tif]

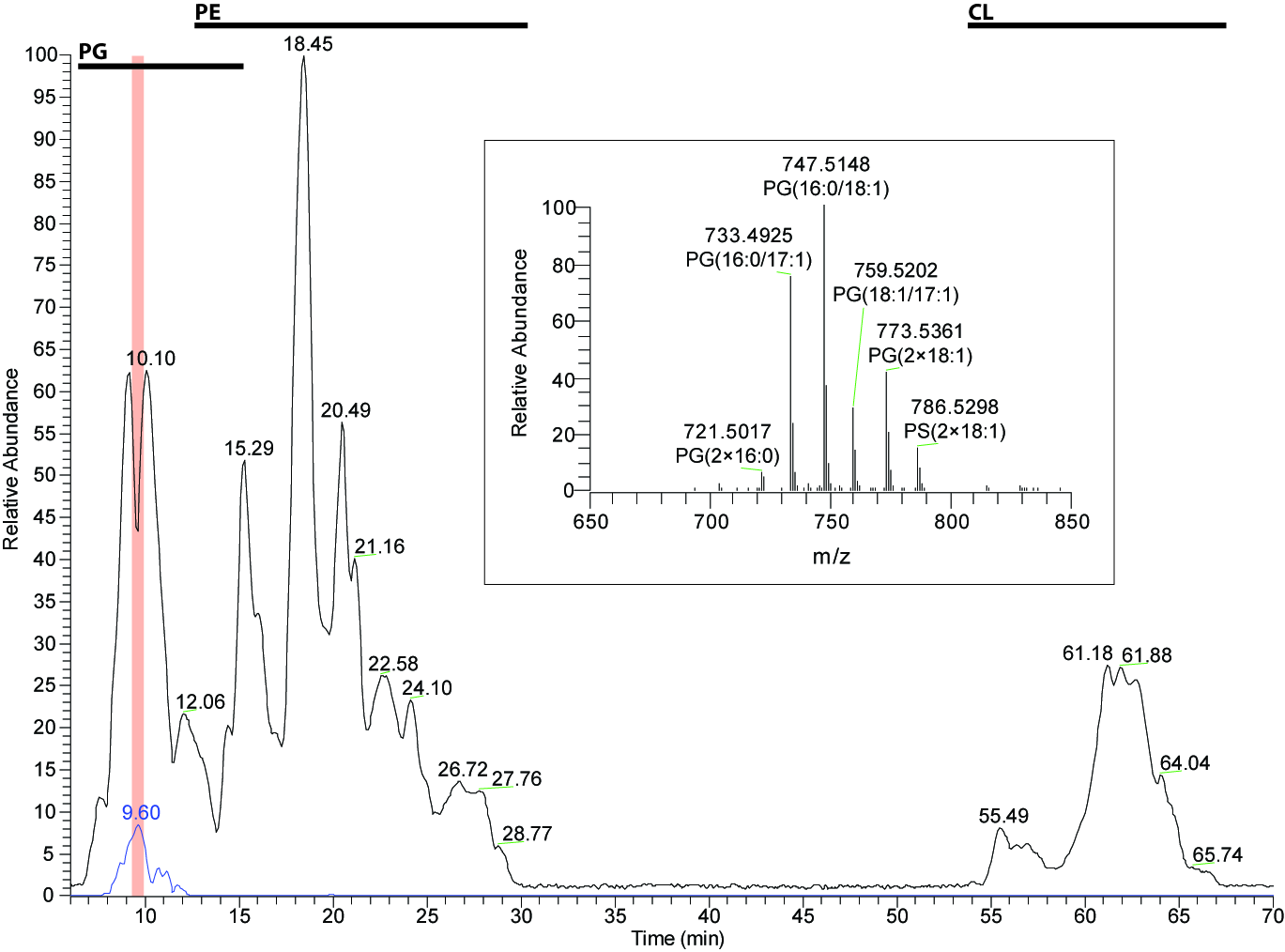

Supplement: Figure S2 — Ion chromatogram of HPLC/MS analysis of a phospholipid extract of Escherichia coli supplemented with 0.1% PS (18∶1/18∶1). Lipids were separated using a BioBasic-4-column as described in “Materials and Methods”. Elution was performed at a flow rate of 50 µL/min by increase of solvent B (70% acetonitrile, 25% 2-propanol, 5% water) vs. solvent A (95% water, 5% acetonitrile). Shown in blue is the trace for the intensity of PS which co-elutes with PG (highlighted by a red bar in the chromatogram). The intervals of the retention times of the individual lipid classes are labeled at the top of the chromatogram. Abbreviations: CL, cardiolipin; PE, phosphatidylethanolamine; PG, phosphatidylglycerol; PS, phosphatidylserine. Inset: Negative ESI-FTICR mass spectrum recorded during the elution of PG classes and PS (18∶1/18∶1). (TIF) [file pone.0042070.s002.tif]

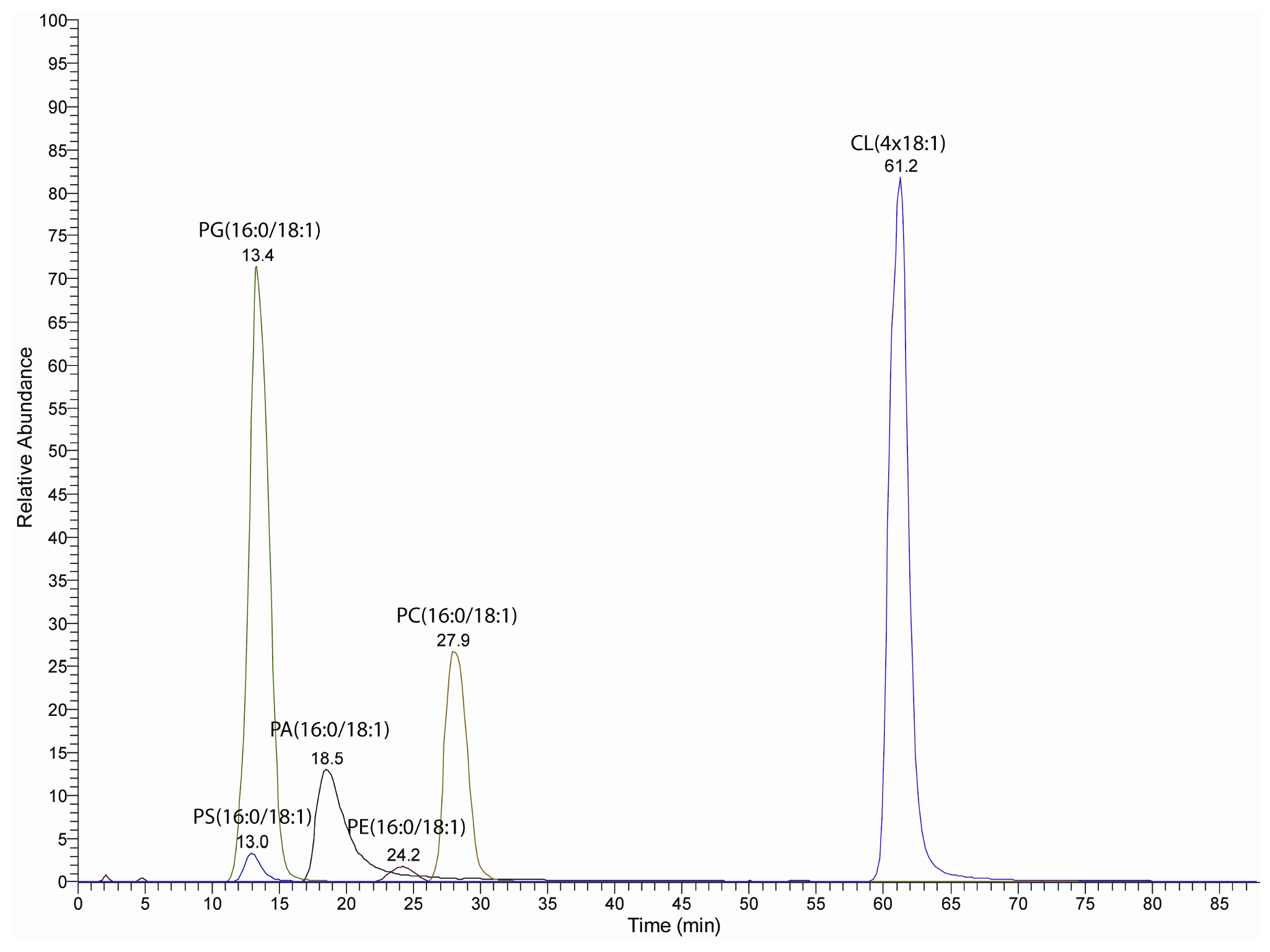

Supplement: Figure S3 — Ion chromatogram of HPLC/MS analysis of an equimolar mixture of the lipid standards PS (16∶0/18∶1), PE (16∶0/18∶1), PC (16∶0/18∶1), PG (16∶0/18∶1), PA (16∶0/18∶1) and CL (4×18∶1). All lipids were used in a concentration of 10 µM in acetonitrile/water/2-propanol/methanol (44.6/36.9/13.5/5, v/v/v/v) and 4 µl were injected into the MS device. Lipids were separated using a BioBasic-4-column as described in “Materials and Methods”. Elution was performed at a flow rate of 50 µL/min by increase of solvent B (70% acetonitrile, 25% 2-propanol, 5% water) vs. solvent A (95% water, 5% acetonitrile). (TIF) [file pone.0042070.s003.tif]

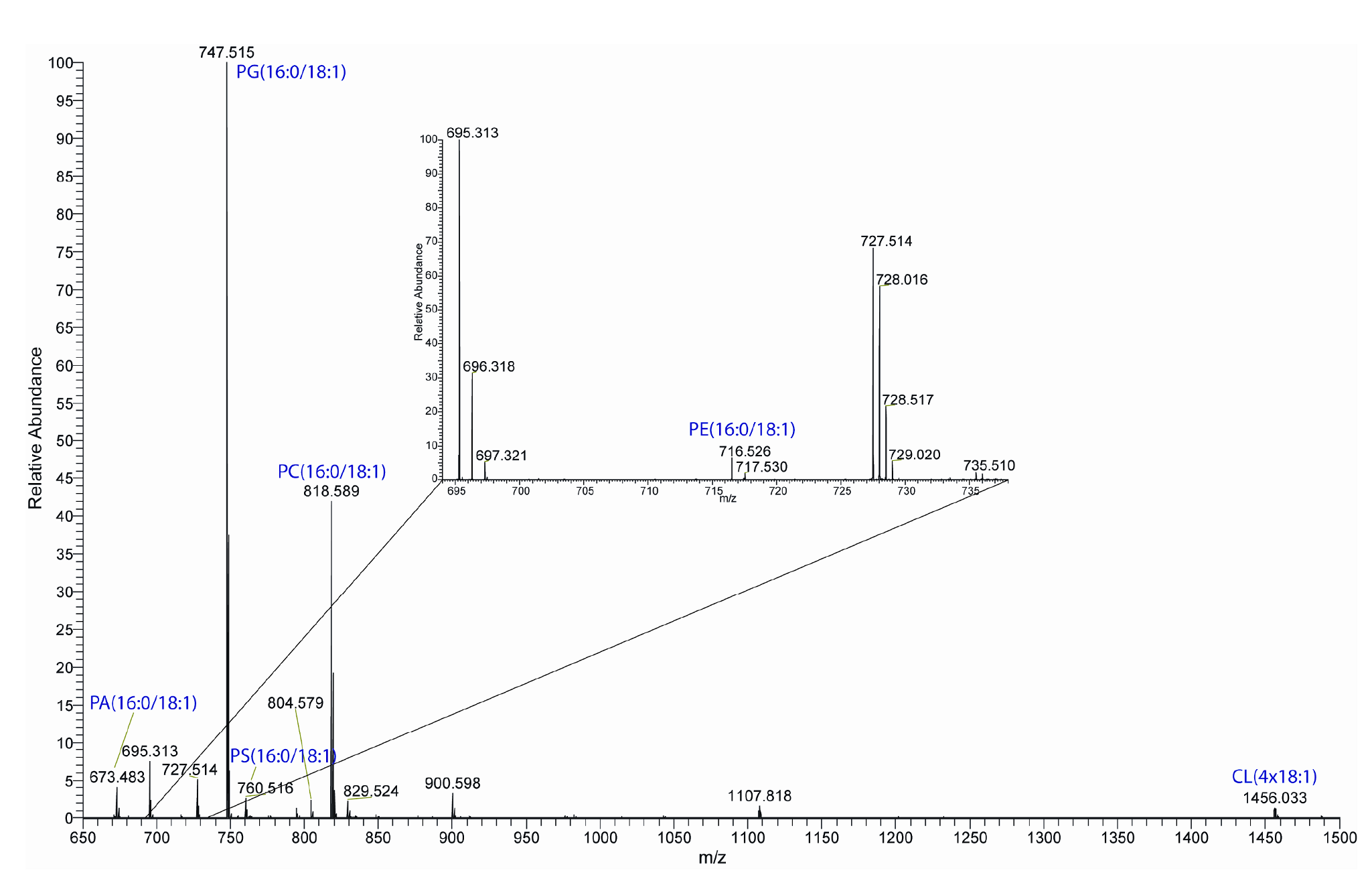

Supplement: Figure S4 — Negative ESI-FTICR mass spectrum of an equimolar mixture of the lipid standards PS (16∶0/18∶1), PE (16∶0/18∶1), PC (16∶0/18∶1), PG (16∶0/18∶1), PA (16∶0/18∶1) and CL (4×18∶1) injected directly. All lipids had a concentration of 1 µM in acetonitrile/water (7/3, v/v). 0.3% triethylamonium acetate was used to enhance ion generation. PC is detected as acetate adduct. Inset: amplified region with the PE species. (TIF) [file pone.0042070.s004.tif]

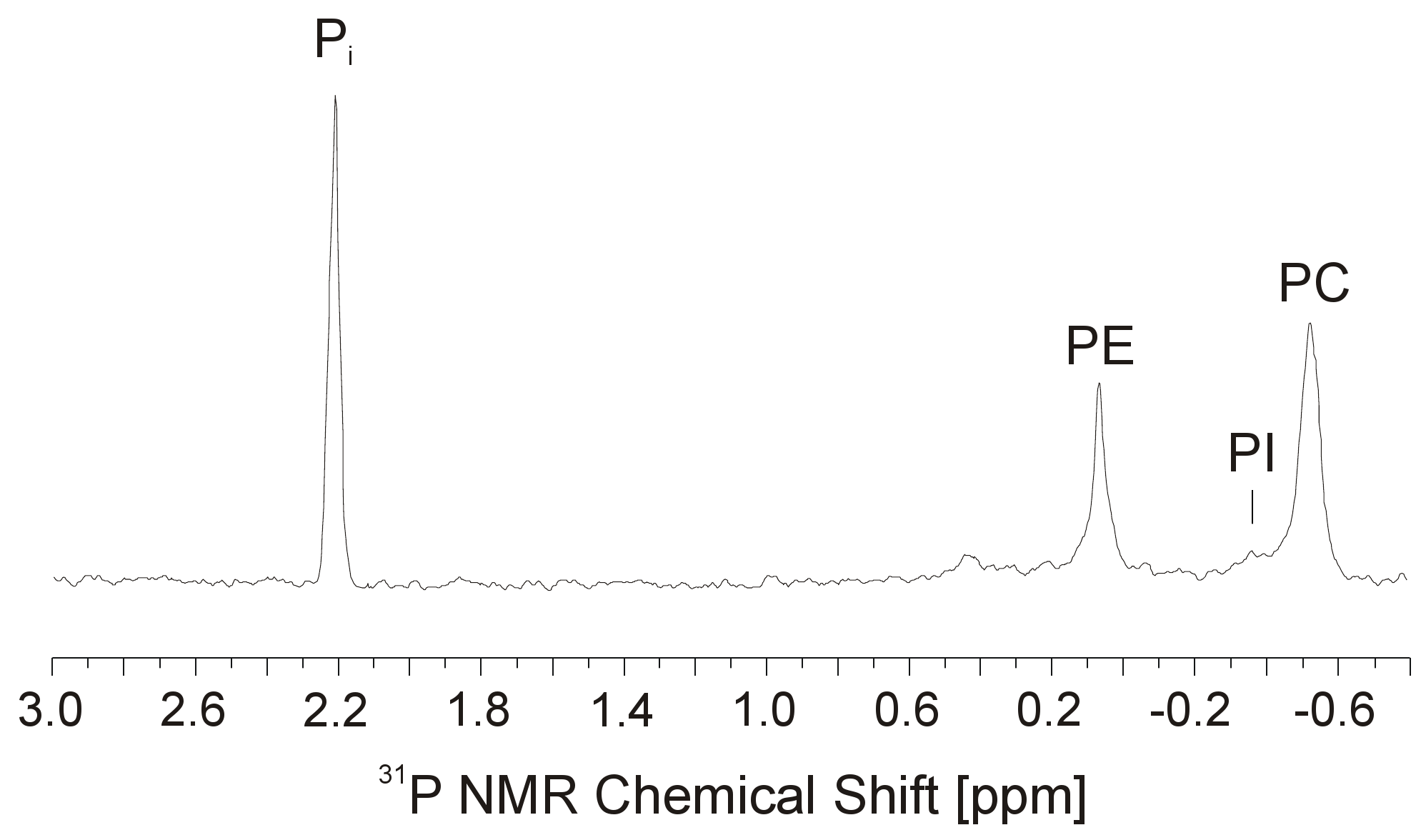

Supplement: Figure S5 — 31P NMR spectra of L. donovani. Parasites were washed and re-solubilized in 200 mM sodium cholate, 5 mM EDTA in D2O. 31P NMR spectra were recorded on a Bruker DRX-600 spectrometer operating at 242.94 MHz. All measurements were performed on 0.6 ml samples in 5 mm NMR tubes using a 5 mm “direct” broadband probe at 37°C. Composite pulse decoupling (Waltz-16) was applied to eliminate 31P-1H coupling. Other NMR parameters were as follows: Data size: 16 k, 60° pulse (5 µs), pulse delay 2 s. A line broadening of 2 Hz was applied for the processing of the free induction decays. Chemical shift assignments were externally referenced relative to 85% orthophosphoric acid at 0.00 ppm. Abbreviations used in peak assignments: PC, phosphatidylcholine; PE, phosphatidylethanolamine; PI, phosphatidylinositol; Pi, inorganic phosphate. (TIF) [file pone.0042070.s005.tif]

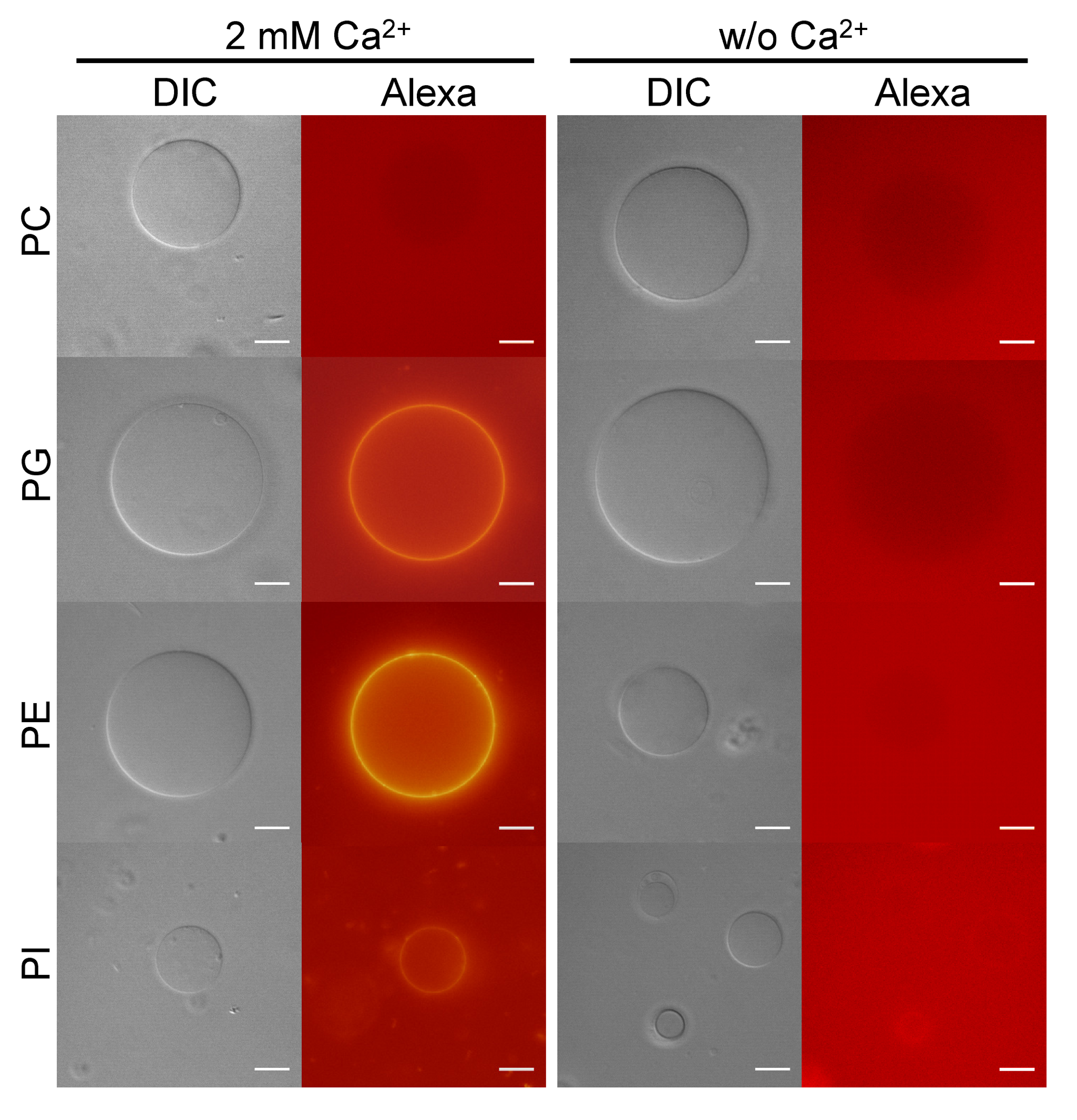

Supplement: Figure S6 — Giant unilamelar vesicles were prepared from different lipids and incubated with annexin V-FITC in the absence of Ca2+ (w/o Ca2+). Vesicles were analyzed by differential interference contrast (DIC) and fluorescence microscopy (FITC). PC: PC (18∶1/18∶1) only; PG: PC (18∶1/18∶1)/PG (18∶1/18∶1), (9/1, mol/mol); PE: PC (18∶1/18∶1)/PE (18∶1/18∶1), (9/1, mol/mol); PI: PC (18∶1/18∶1)/PI, (9/1, mol/mol). Bar, 10 µm. (TIF) [file pone.0042070.s006.tif]

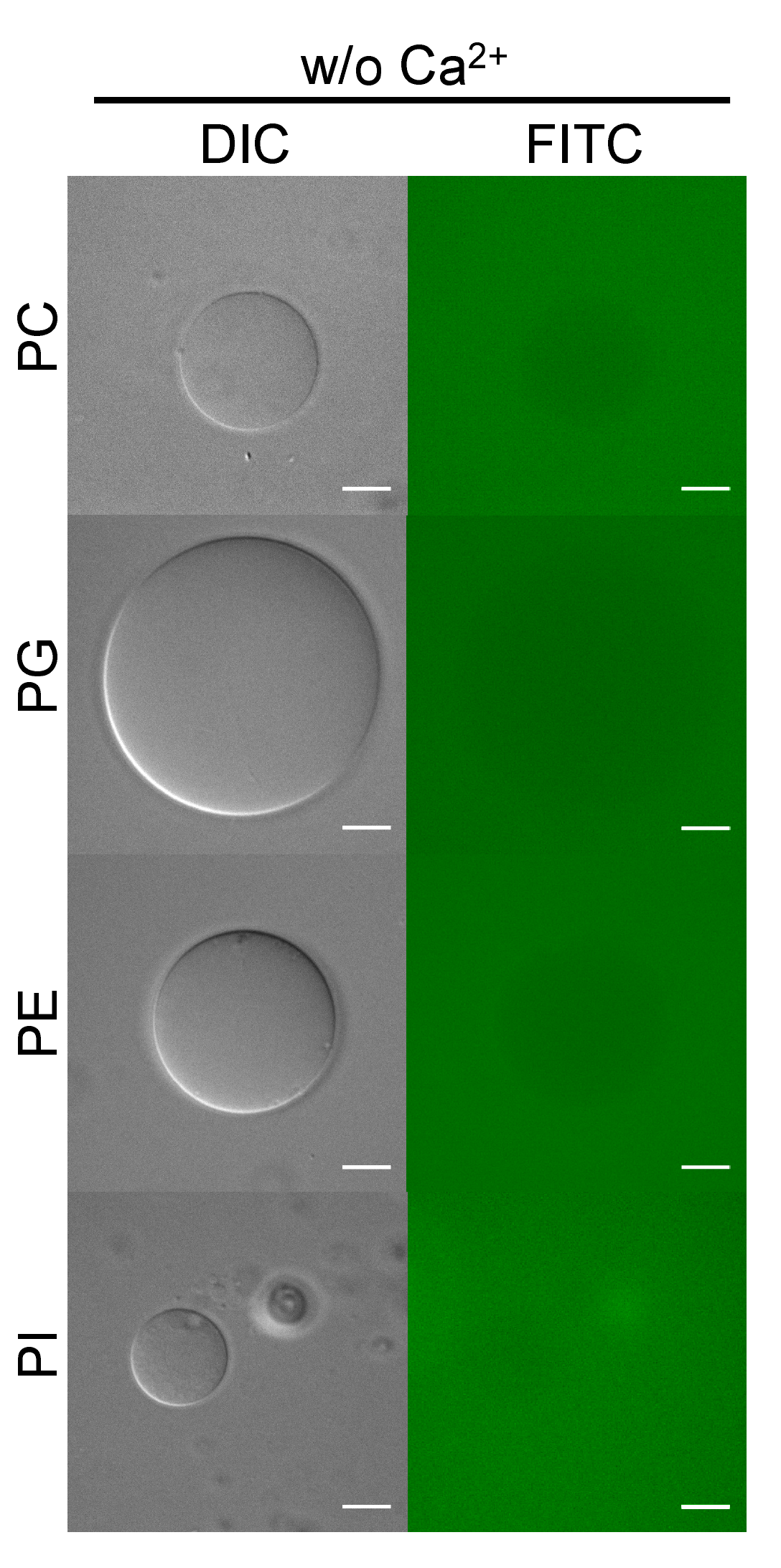

Supplement: Figure S7 — Giant unilamelar vesicles were prepared from different lipids and incubated with Annexin V-Alexa 568 (2 µL/mL; Roche Diagnostics, Mannheim, Germany) in the presence or absence of Ca2+. Vesicles were analyzed by differential interference contrast (DIC) and fluorescence microscopy (Alexa; excitation band-pass filter 515–560, beam splitter 580, emission long-pass filter 590). PC: PC (18∶1/18∶1) only; PG: PC (18∶1/18∶1)/PG (18∶1/18∶1), (9/1, mol/mol); PE: PC (18∶1/18∶1)/PE (18∶1/18∶1), (9/1, mol/mol); PI: PC (18∶1/18∶1)/PI, (9/1, mol/mol). Bar, 10 µm. (TIF) [file pone.0042070.s007.tif]
