# Supplementary material for: Leishmania Promastigotes Lack Phosphatidylserine but Bind Annexin V upon Permeabilization or Miltefosine Treatment
Source: PLoS One. 2012 Aug 1;7(8):e42070. doi: 10.1371/journal.pone.0042070 (PMC3411662; doi:10.1371/journal.pone.0042070)
Supplement: Table S1 — HPLC/MS analysis of a lipid extract of L. amazonensis. (DOC) [file pone.0042070.s008.DOC]

**Supplementary Table S1.** HPLC/MS analysis of a lipid extract of *L. amazonensis*

| **Phospholipid^a^** | **Retention Time (min)^b^** | **Relative abundance (%)^c^** |
| --- | --- | --- |
| lyso-PI (18:1) | 1.9 | Only this species detectable |
| lyso-PE(18:2) | 2.9 | Only this species detectable |
| lyso-PC(18:3) | 3.3 | 17.8 |
| lyso-PC(18:2) | 3.6 | 67.1 |
| lyso-PC(18:1) | 4.1 | 9.9 |
| lyso-PC(18:0) | 5.1 | 5.2 |
| PC(18:2/22:6) | 16.5 | 19.1 |
| PC(2×18:2) | 18.1 | 36.1 |
| PC(18:2/18:1) | 22.7 | 11.3 |
| p-PC(18:1a/18:2) | 33.0 | 34.7 |
| p-PC(18:0a/18:2) | 35.5 | 65.3 |
| IPC(t16:0/18:0) | 4.4 | 20.3 |
| IPC(d16:1/18:0) | 5.0 | 58.2 |
| IPC(d17:1/18:0) | 5.5 | 8.2 |
| IPC(d18:1/18:0) | 6.6 | 13.3 |
| PE(18:3/18:2) | 11.2 | 9.1 |
| PE(2×18:2) | 13.4 | 36.3 |
| PE(18:2/18:1) | 16.9 | 28.9 |
| PE(18:0/18:2) | 21.9 | 17.4 |
| PE(18:0/18:1) | 26.3 | 8.3 |
| p-PE(16:1a/18:2) | 18.4 | 7.8 |
| p-PE(17:1a/18:2) | 20.8 | 7.0 |
| p-PE(18:1a/18:2) | 24.6 | 69.0 |
| p-PE(18:1a/18:1) | 29.7 | 9.8 |
| PI(18:2/18:0) | 8.5 | 18.8 |
| PI(18:1/18:0) | 10.6 | 79.2 |
| p-PI(18:0a/18:2) | 10.1 | 20.9 |
| p-PI(18:0a/18:1) | 12.2 | 79.1 |
| CL(22:6/18:2, 22:6/18:2) | 54.6 | 15.7 |
| CL(22:6/18:2, 22:6/22:5) | 55.0 | 18.6 |
| CL(22:6/18:2, 22:5/22:5) | 55.6 | 18.3 |
| CL(22:6/18:2, 22:5/18:2) | 56.4 | 21.3 |
| CL(22:6/18:1, 22:5/22:5) | 56.5 | 11.4 |

^a^lyso-PE, lyso-phosphatidylethanolamine; lyso-PC, lyso-phosphatidylcholine; PC, phosphatidylcholine; p-PC, plasmalogen phosphatidylcholine; IPC, inositolphosphorylceramide; PE, phosphatidylethanolamine; p-PE, plasmalogen phosphatidylethanolamine; PI, phosphatidylinositol; p-PI, plasmalogen phosphatidylinositol; CL, cardiolipin. The letter “a” denotes an alkyl- or alkenyl-ether residue in the plasmalogen species. In all cases only the most abundant fatty acyl compositions are indicated, while the detailed evaluation of very minor species was not the subject of this paper. The inositolphosphorylceramides are denoted as follows: IPC (long chain base/fatty acyl residue) with the prefixes “d” and “t” to designate di-and trihydroxy species.

^b^Retention time of HPLC.

^c^Percentage within the given phospholipid class as determined from the MS-signal intensities. The fatty acyl compositions were assigned to the *sn*-1 and *sn*-2 positions by means of the differing signal intensities in the fragment ion spectra.
